# Single and Binge Methamphetamine Administrations Have Different Effects on the Levels of Dopamine D2 Autoreceptor and Dopamine Transporter in Rat Striatum

**DOI:** 10.3390/ijms15045884

**Published:** 2014-04-08

**Authors:** Heli Chauhan, Bryan A. Killinger, Cheryl V. Miller, Anna Moszczynska

**Affiliations:** Department of Pharmaceutical Sciences, Eugene Applebaum College of Pharmacy and Health Sciences at Wayne State University, 259 Mack Ave, Detroit, MI 48201, USA; E-Mails: helichauhan@wayne.edu (H.C.); bakillinger@wayne.edu (B.A.K.); dy0179@wayne.edu (C.V.M.)

**Keywords:** dopamine D2 receptor, dopamine transporter, methamphetamine, striatum, *in vivo*

## Abstract

Methamphetamine (METH) is a central nervous system psychostimulant with a high potential for abuse. At high doses, METH causes a selective degeneration of dopaminergic terminals in the striatum. Dopamine D2 receptor antagonists and dopamine transporter (DAT) inhibitors protect against neurotoxicity of the drug by decreasing intracellular dopamine content and, consequently, dopamine autoxidation and production of reactive oxygen species. *In vitro*, amphetamines regulate D2 receptor and DAT functions via regulation of their intracellular trafficking. No data exists on axonal transport of both proteins and there is limited data on their interactions *in vivo*. The aim of the present investigation was to examine synaptosomal levels of presynaptic D2 autoreceptor and DAT after two different regimens of METH and to determine whether METH affects the D2 autoreceptor-DAT interaction in the rat striatum. We found that, as compared to saline controls, administration of single high-dose METH decreased D2 autoreceptor immunoreactivity and increased DAT immunoreactivity in rat striatal synaptosomes whereas binge high-dose METH increased immunoreactivity of D2 autoreceptor and had no effect on DAT immunoreactivity. Single METH had no effect on D2 autoreceptor-DAT interaction whereas binge METH increased the interaction between the two proteins in the striatum. Our results suggest that METH can affect axonal transport of both the D2 autoreceptor and DAT in an interaction-dependent and -independent manner.

## 1. Introduction

Dopamine (DA) receptors belong to the super-family of membrane-bound proteins, termed G-protein coupled receptors. Stimulation of D1-like receptors (D1 and D5) activates adenylyl cyclase and increases the production of cAMP. In contrast, stimulation of D2-like receptors (D2, D3 and D4) inhibits adenylyl cyclase and cAMP production [1]. Two main different splice variants of D2 receptor are known in humans and rodents: the D2short (D2S) and D2long (D2L) built of 414 and 443 amino acids, respectively [2–4]. The difference in the length of the splice variants causes a difference in the length of the third intracellular loop. Activation of D2S receptors, which are presynaptic autoreceptors, inhibits DA synthesis and stimulation-dependent DA release [5–7]. The D2S receptors also regulate the function of dopamine transporter (DAT), a membrane protein regulating dopaminergic (DAergic) neurotransmission by re-uptake of released DA [8–10].

Methamphetamine (METH) is a widely abused psychostimulant that causes neurotoxicity to DAergic nerve terminals in experimental animals and humans when administered at high doses [11]. D2 receptor antagonists and DAT blockers were shown to attenuate METH-induced degeneration of DAergic terminals [12–14] thus implicating D2 receptors as well as DAT in mediating the neurotoxicity of the drug. METH is initially taken up to DAergic terminals via the DAT. Once in the terminal, it increases DA efflux from the storage vesicles and causes a release of DA into the synaptic cleft by reversing DAT function [15,16]. Both DA effluxes, intra-terminal and extra-terminal, have been shown to mediate METH neurotoxicity; the increase in intracellular DA is followed by autoxidation of DA and oxidative damage to proteins and lipids within DAergic terminals [11]. DAT blockers might protect against METH toxicity via attenuation of METH uptake into DAergic terminals [17] whereas D2 receptor antagonists might protect via reduction of releasable pool of DA within the storage vesicles as well as via interaction with DAT and inhibition of DAT-mediated reuptake of DA [12].

Amphetamines regulate D2 receptor and DAT functions at the plasma membrane via regulation of their intracellular trafficking. *In vivo*, amphetamines cause internalization of D2 receptors indirectly, via a release of DA into the synaptic cleft and activation of the receptors [18–20]. Amphetamines administered *in vitro* (to cultured cells or to isolated synaptosomes) can mobilize DAT to the plasma membrane or trigger its internalization, depending on the duration of drug exposure and on activation of D2S receptors [21]. Only two studies examined intracellular trafficking of striatal DAT after *in vivo* administration of amphetamines [22,23] and produced results conflicting with the results obtained *in vitro*. METH neurotoxicity shares several molecular mechanisms of neurodegeneration with Parkinson’s disease (PD) [24], suggesting that an impairment of axonal transport found in PD [25] might also be a feature of METH neurotoxicity. To our knowledge, no studies examined axonal trafficking of striatal D2S and DAT after systemic METH administration. *In vitro*, expression of DAT at the plasma membrane is regulated by D2S receptor indirectly with the mediation of protein kinase Cβ [26] and directly via protein-protein interaction. As there is very limited data on DAT-D2S interaction *in vivo* [27,28] further investigation of the interaction is warranted.

The aims of the present study were the following: (i) to examine the effects of *in vivo* METH administration on D2S and DAT axonal transport in the nigrostriatal DA pathway (by measuring the levels of D2 receptor and DAT in striatal synaptosomes) and (ii) to determine whether *in vivo* METH administration affects D2S receptor-DAT interaction in striatal DAergic terminals. We employed two METH regimens: administration of single high dose of METH (short-term exposure) and binge METH administration (long-term exposure), which is a well-established neurotoxic regimen.

We have determined that, as compared to saline controls, the immunoreactivity of D2S receptor in rat striatal synaptosomes is decreased whereas immunoreactivity of DAT is increased at 10 min and 5 h, respectively, after a single injection of METH. Multiple injections of METH have no effect on DAT immunoreactivity but they increase D2S receptor immunoreactivity in striatal synaptosomes at 1 h after the last injection of the drug. We have also determined that single METH, non-toxic to DAergic terminals, does not affect D2 receptor-DAT interaction whereas neurotoxic binge METH increases the interaction between the two proteins. Our results demonstrate that single and binge METH administrations have different effects on the levels of dopamine D2S receptor and DAT in the rat striatum and suggest that METH can affect axonal transport of both the D2S and DAT in a D2S-DAT interaction-dependent and -independent manner.

## 2. Results and Discussion

### 2.1. Dopamine D2 Receptor and DAT Species in Rat Striatal Synaptosomes

**Figure 1.**
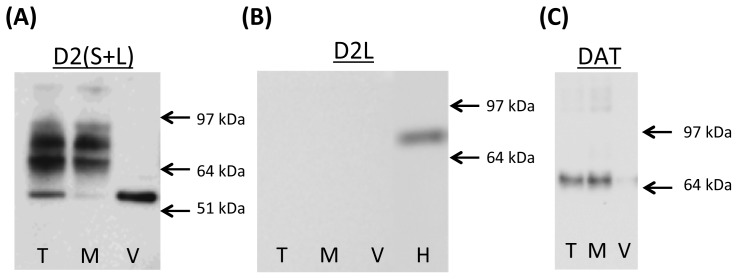
Dopamine D2 receptor and dopamine transporter (DAT) species in rat striatal synaptosomes. (**A**) The immunoreactivity of the antibody recognizing both D2S and D2L isoforms of D2 receptor; (**B**) the immunoreactivity of the antibody recognizing the D2L receptor; and (**C**) the immunoreactivity of the antibody recognizing the DAT. Abbreviations: D2L, D2long receptor; D2S, D2short receptor; H, homogenate; M, membrane synaptosomal fraction; T, total synaptosomal fraction; V, vesicular synaptosomal fraction.

The D2 receptor antibody from EMD Millipore Corp. (Billerica, MA, USA) recognizes both D2S and D2L receptor. To determine to what extent post-synaptic D2L receptors “contaminate” our synaptosomal preparations, synaptosomal fractions were loaded on gels and the membranes were probed with D2(S+L) or D2L antibody. D2(S+L) antibody produced 3 main bands at ~68, ~75 and ~90 kDa as well as 2 weaker bands, ~53 and ~110 kDa band (Figure 1A). The D2L antibody detected a band at ~75 kDa in striatal homogenates but not in striatal synaptosomes (Figure 1B). Western blotting with antibody against DAT revealed one band of a molecular weight of ~70 kDa (Figure 1C).

The mature D2S receptor exists in 2 isoforms, unglycosylated and glycosylated [4,29,30]. Similarly, the mature DAT exists in 2 isoforms, unglycosylated and glycosylated [31]. Both proteins are known to form homo- and hetero-dimers and oligomers [10,30–33]. Both receptors undergo glycosylation in the Golgi apparatus and travel to dendrites and distal terminals in this form; therefore, we did not expect to detect the unglycosylated forms in synaptosomal preparations. The expected molecular weight for unglycosylated D2L and D2S receptor is ~44 and ~41 kDa, respectively, judging from their amino acid sequence. They appear slightly higher on gels if post-translationally modified at functional groups. No bands around these molecular weights were detected, indicating that, as expected, our synaptosomal preparations do not contain detectable levels of unglycosylated D2 receptor (D2R; D2short or D2long) or DAT. Crude synaptosomal preparations contain a number of dendritic synaptosomes, which could contribute glycosylated D2L immunoreactivity to our blots. The mature rat D2S receptor exists mainly in the unglycosylated and highly glycosylated form (~70–90 kDa) whereas D2L exists also in an intermediate, partially glycosylated form [4,29,34]. We did not detect D2L immunoreactivity >70 kDa in our preparations when using D2L receptor antibody (Figure 1B), which suggested that the D2L receptor was at negligible levels in our synaptosomal preparations. This notion was supported by minimal D2 receptor immunoreactivity in cytosol-vesicular fractions where D2L receptor would be expected [35]. However, we detected a band of ~53 kDa using D2(S+L) antibody, which was the most abundant in the vesicular-cytosolic fraction. This band could be the partially glycosylated D2L from cytosol of dendritic synaptosomes recognizable by D2(S+L) but not D2L antibody or an unspecific band [4,34]. It is important to differentiate between D2S and D2L receptor because post-synaptic D2L might introduce confounding evidence to data interpretation if its responses to METH are different than those of D2S receptor. Differentiation between the isoforms allowed examination of METH effects on D2S separately from the effects of METH on D2L. In addition to unglycosylated and glycosylated D2 receptor monomers, bands at higher molecular weight have been reported [29,30]. In agreement with these studies, the D2 receptor antibody detected three main bands at ~68, ~75, and ~90 kDa as well as a weak band at ~110 kDa. The 75 and 90 kDa bands likely represent D2S receptor at different levels of glycosylation whereas ~110 kDA band likely represents D2S receptor complexes with itself or other proteins [20,29,33], e.g., with sGi2 [20]. In agreement with previous reports of mainly plasma membrane localization of the DAT [23,31], the glycosylated form of DAT was detected exclusively in the membrane-endosomal fraction at 70 kDa; weak higher molecular weight bands were also observed.

### 2.2. Single High Dose of Methamphetamine Causes Transient Hyperthermia but Does not Result in Neurotoxicity to Dopaminergic Terminals

**Figure 2.**
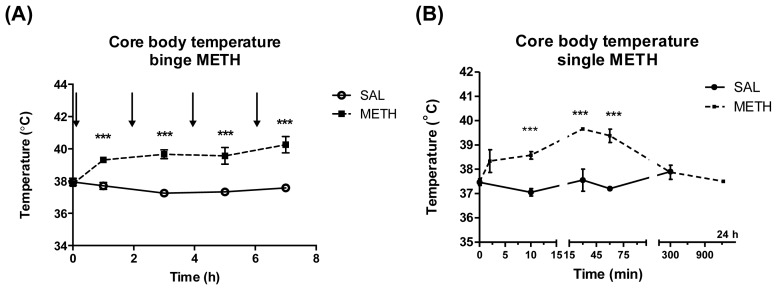
The effect of binge and single dose METH on core body temperature. (**A**) In the binge dose regimen, core body temperatures (°C) were measured before the first METH or saline injection and 1 h after each METH or saline injection. Values are expressed as mean ± SEM. The arrows indicate METH injections. Binge METH caused hyperthermia lasting for at least 6 h; (**B**) in the single dose regimen, core body temperatures were measured immediately before sacrifices. Single injection of METH caused transient hyperthermia at 10, 30, and 60 min that peaked at 30 min. *** Significant difference (*p* < 0.001) between saline and METH. Abbreviations: METH, methamphetamine; SAL, saline.

Signs of METH neurotoxicity to DAergic terminals include persistent decreases in DAergic markers measured at 3 d or later after METH [11,36]. The decreases start to manifest themselves at 24 h; however, they often do not reach the maximum at this time point [37,38]. Binge high-dose METH administration is an established model of METH neurotoxicity producing 40%–60% decreases in the levels of DA, its metabolites, and other DAergic markers at 3–7 d after the drug [11,39]. METH-induced hyperthermia is an important contributor to the neurotoxicity [11]; therefore, core body temperatures were recorded during both METH regimens. As in our previous study [39], binge METH administration (4 × 10 mg/kg, every 2 h, i.p.) resulted in statistically significant increases in core body temperatures as compared to saline controls (39.3–40.2 *vs.* 37.5–37.9 °C) (two-way ANOVA (Analysis of Variance) with repeated measures followed by Student-Neuman-Keuls *post hoc* test; *n* = 11/group) (Figure 2A). There was a significant main effect of the treatment (SAL (saline) or METH) (*F*_(1,20)_ = 44.39, *p* < 0.0001) and time (*F*_(4,80)_ = 5.24, *p* < 0.001) as well as significant treatment *x* time interaction (*F*_(4,80)_ = 12.25, *p* < 0.001). Single METH injection (6 mg/kg, i.v.) also induced an increase in core body temperature over time that peaked at 30 min (39.7 °C) and was statistically significant at 10, 30, and 60 min (Figure 2B). There was a significant main effect of the treatment (SAL or METH) (*F*_(1,47)_ = 34.10, *p* < 0.0001) and time (*F*_(6,47)_ = 6.07, *p* < 0.001) as well as significant treatment *x* time interaction (*F*_(6,47)_ = 7.02, *p* < 0.001). The temperature was at the control level at 5 h. Single 6 mg/kg dose of METH administered intravenously (i.v.) (closely corresponding to single 10 mg/kg MEH dose administered by intraperitoneal (i.p.) injection due to higher bioavailability of METH after i.v. than i.p. route; see the Experimental Section) was not neurotoxic to DAergic terminals as assessed by the levels of DA at 7 d after the last METH injection (saline: 14.7 ± 0.4, METH: 14.7 ± 0.5 ng/mg tissue, *p* = 0.998, unpaired Student’s *t*-test) despite induction of hyperthermia. This was an expected result, as METH neurotoxicity occurs after regimens that result in a relatively long presence of METH in the brain, such as multiple injections of the drug (4 × 5–10 mg/kg) or a massive single dose of METH (30–60 mg/kg) [11]. Binge and massive single doses of METH induce hyperthermia that lasts longer than 4 h [40,41]; therefore, our data suggest that short-lasting hyperthermia does not lead to neurotoxicity to DAergic terminals.

### 2.3. Methamphetamine Differentially Affects Dopamine D2S Receptor and DAT Immunoreactivity in Rat Striatal Synaptosomes

To determine whether single *vs.* binge high-dose METH affects the levels of pre-synaptic D2 receptor and DAT in striatal synaptosomes (representing axonal terminals), rats were treated with binge METH (4 × 10 mg/kg, every 2 h, i.p.) or saline (1 mL/kg) and sacrificed at 1 or 24 h after the last injection. Amphetamines and DA can affect trafficking of DAT in a bimodal manner, depending on the length of the exposure [21]. D2 receptor usually internalizes upon exposure to DA; however, it can also be mobilized to the plasma membrane [42]. Some of these effects are very rapid and transient [43–45]. To examine the effects of a single dose METH (and METH-released DA) on D2S and DAT over time, another group of rats was treated with 6 mg/kg i.v. METH or saline and sacrificed at 2 min, 10 min, 30 min, 1 h, 5 h, and 24 h after the injection. We chose the i.v. and not i.p. route for administration of a single dose METH because the i.v. route allows for faster accumulation of METH in the brain than the i.p. route [46–48], thus allowing monitoring of rapid and transient trafficking of the membrane proteins. We chose it also to decrease inter-individual variation as absorption from the peritoneal cavity may vary depending on the amount of food in the gut and needle placement. Bioavailability of METH after i.p. injection is ~60% of its availability after i.v. injection [49]; therefore, we chose 6 mg/kg of METH for the i.v. route. Striatal synaptosomal fractions (total, membrane-endosomal and cytosol-vesicular fraction) were examined for D2S receptor, D2L receptor, and DAT immunoreactivity (between 64 and 97 kDa).

#### 2.3.1. Single Dose of Methamphetamine Decreases the Immunoreactivity of D2S Receptor in Rat Striatal Synaptosomes

**Figure 3.**
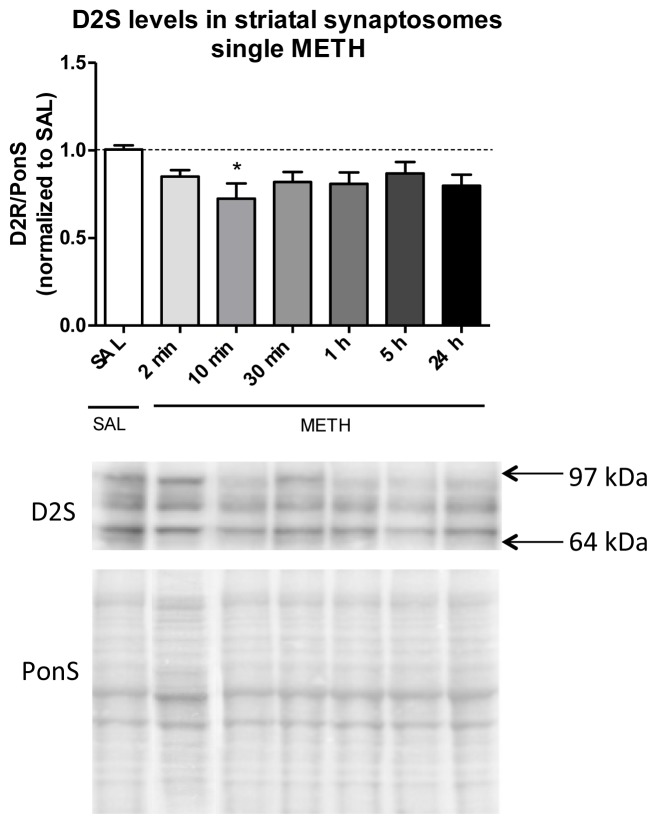
The effect of single intravenous (i.v.) dose of METH on the immunoreactivity of dopamine pre-synaptic D2 autoreceptor (D2S) in rat striatal synaptosomes (total fraction). The time course of D2S receptor immunoreactivity after 6 mg/kg METH shows a decrease (−28%, * *p* < 0.05) in D2S signal at 10 min after METH injection. Abbreviations: METH, methamphetamine; PonS, Ponceau S; SAL, saline.

To study the effects of single METH on the levels of D2S receptor in DAergic terminals over 24 h, rats were injected intravenously with one dose of METH (6 mg/kg) or saline and sacrificed at several time points after the treatment. Striatal synaptosomes were examined for D2(S+L) and D2L immunoreactivity. As compared to the control group, D2(S+L) immunoreactivity in the total synaptosomal fraction was significantly decreased at 10 min after METH (−28%, * *p* < 0.05, *n* = 5–9 rats/group; one-way ANOVA followed by the Dunnett’s multiple comparison *post hoc* test; *F*_(6,32)_ = 2.3, *p* = 0.059, *n* = 3–9 rats/group) (Figure 3). The membranes probed with D2L antibody did not produce immunoreactivity (not shown), indicating that the decrease in D2(S+L) immunoreactivity represents a decrease in D2S receptor immunoreactivity.

Amphetamines increase extracellular DA levels in experimental animals and humans [11]. Previous *in vivo* studies have found that increasing endogenous DA levels in the striatum using single amphetamine injection leads to a decrease in radioligand binding to striatal D2 receptors [18–20]. The decrease involves internalization of D2 receptors into an intracellular compartment(s). For example, single s.c. injection of amphetamine led to a rapid (within 30 min) internalization of D2 receptor and its redistribution from the cell surface to endosomes and also to other intracellular compartment(s); the decrease lasted up to 6 h after amphetamine [18]. Another study in rat striatum has demonstrated that activation of D2 receptors by an agonist or DA results in decreased D2S and D2L receptor levels at the plasma membrane and that the effect is mediated by sGi2 [20]. In agreement with these results, we found a rapid (within 10 min) decrease in D2 receptor immunoreactivity in total synaptosomal fraction after single dose METH relative to saline controls; the decrease appeared transient (Figure 3). Lack of D2L immunoreactivity suggested a lack of contribution of post-synaptic D2L receptor to the decrease; however, contribution of the D2L receptor cannot be totally excluded due to a potential lower binding affinity of D2L antibody *vs.* D2(S+L) antibody to the D2L receptor. The study of Sun and colleagues [18] did not differentiate between D2S and D2L receptor internalization; therefore, the isoform that stayed internalized for 6 h might have been D2L receptor. In support of our finding, the results from their study suggested that portion of D2S might have been transported out of the DAergic terminals in addition to being internalized and packed into the endosomes.

Our study did not differentiate between plasma membrane and endosomes; therefore we do not know whether, or to what extent, the D2S receptor trafficked from the plasma membrane to endosomal compartments. Our study demonstrated a small decrease in total levels of D2S receptor after METH, suggesting that a small population of D2S receptors might have been “removed” from DAergic terminals via increased transport of D2S receptor out of the terminal and/or decreased mobilization of D2S receptor to the terminal. An alternative explanation for the decrease in synaptosomal immunoreactivity of the D2S receptor is increased degradation of the receptor, either as an adaptive response to DA release [50] or due to oxidative damage to the receptor [51]. Increased degradation seems unlikely, taking into account the fact that the decrease was observed as soon as 10 min after METH injection. A more plausible explanation is oxidative stress-induced modification to the *N*-terminus of D2S [52]. Potentially, the decrease in D2S levels was triggered by increased co-internalization of D2 receptor-adenosine A2A complexes [32,53].

Amphetamines have low affinity for D2 receptors. Therefore, the decrease in D2S immunoreactivity was more likely triggered by METH-released extracellular DA rather than METH itself. Activation of pre-synaptic D2S autoreceptors by DA negatively regulates DA synthesis and DA release [5–7] whereas inactivation of the autoreceptors decreases DAT function and vesicular DA content [12]. The decrease in D2S levels after single i.v. METH might represent removal of D2S receptors form the plasma membrane and terminal to regulate one of the latter events. DA lasts in the synaptic cleft about 2 h after single medium-dose i.v. amphetamine [54]. Consequently, steady return of the D2 receptor immunoreactivity to the control values (Figure 3) might represent, in part, recovery of its axonal transport. In summary, our finding suggests that DA might play a role in trafficking of the D2S receptor in and out of the DAergic terminal.

#### 2.3.2. Single Dose of Methamphetamine Increases the Immunoreactivity of DAT in Rat Striatal Synaptosomes

**Figure 4.**
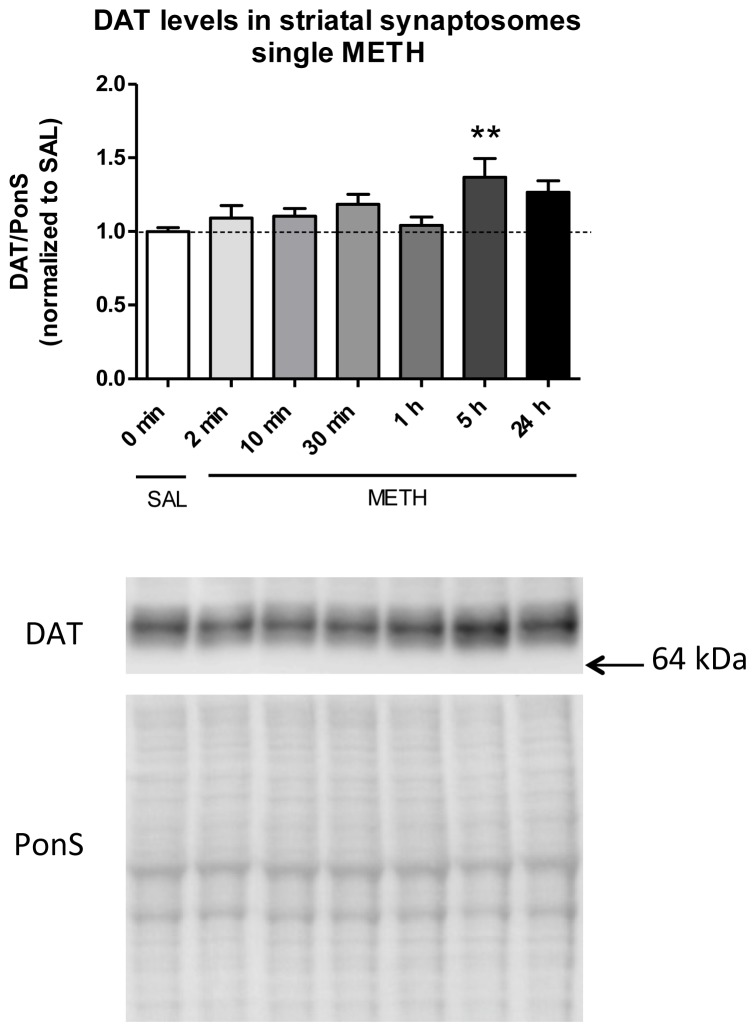
The effect of single intravenous (i.v.) dose of METH on the immunoreactivity of dopamine transporter (DAT) in rat striatal synaptosomes (total fraction). The time course of DAT immunoreactivity after 6 mg/kg METH shows an increase (+37%, ** *p* < 0.01) in DAT immunoreactivity at 5 h after METH injection. Abbreviations: METH, methamphetamine; PonS, Ponceau S; SAL, saline.

To study the effects of a single dose of METH on the levels of DAT in DAergic terminals over 24 h, rats were injected intravenously with one dose of METH (6 mg/kg) or saline and sacrificed at several time points after the treatment. Striatal synaptosomes were examined for DAT immunoreactivity. As compared to the control group, DAT immunoreactivity in total synaptosomal fraction was significantly increased at 5 h after METH (+37%, ** *p* < 0.01, *n* = 8–9 rats/group; one-way ANOVA followed by the Dunnett’s multiple comparison *post hoc* test; *F*_(6,60)_ = 2.7, *p* < 0.05, *n* = 4–17 rats/group) (Figure 4).

*In vitro* studies have demonstrated that amphetamines very rapidly (within 1–2 min) and transiently mobilize DAT from the cytosol or endosomes to the plasma membrane and cause its internalization later on [22,43,55–57]. Our finding does not agree with the results from these studies. We did not observe rapid and transient increase in DAT immunoreactivity or DAT internalization. Instead, we observed a small but steady increase in DAT immunoreactivity over 5 h. In studies with synaptosomes, DAT was mobilized to the plasma membrane from the endosomes, not from a location outside of the terminal. We did not measure DAT immunoreactivity between intracellular fractions; therefore, mobilization of DAT to the plasma membrane from the endosomes cannot be excluded. Alternatively, despite efforts to catch potential very early METH-induced changes to DAT axonal transport, we did not detect them, or they did not occur.

Our finding is novel in that it suggests that METH might affect DAT trafficking along the axons. Thus, the observed increase might be due to increased anterograde transport and/or decreased retrograde transport of the DAT. Our results largely agree with two studies that, like our study, administered amphetamines *in vivo*. They reported lack of change in DAT immunoreactivity in all synaptosomal fractions at 5, 15, 30 or 60 min after injection of 15 mg/kg amphetamine (s.c.) and in the membrane fraction at 45 min after injection of 2 mg/kg amphetamine (i.p.) as compared to controls [22,23]. Both studies did not examine DAT immunoreactivity beyond 1 h and we observed a significant increase in DAT only at 5 h, not earlier (Figure 4). As both studies showed lack of change in DAT immunoreactivity up to 1 h after METH despite different doses and routes of METH administration and, consequently, slightly different METH pharmacokinetics [47,49], the results from these studies suggest that dose and route of drug administration do not affect axonal transport of DAT within this time frame.

Most of the previous studies on DAT trafficking addressed intracellular trafficking of the transporter with METH or DA added to cultured cells or isolated striatal synaptosomes. In these studies, axonal transport, post-synaptic signaling of DA, and combined action of DA and METH on the DAT did not play a role in regulation of DAT. In addition, studies in cell cultures did not take into account the fact that DAT traffics differently in different brain areas [58]. Our subcellular fractionation technique does not allow separation of plasma membrane and endosomal cargo. Therefore, we do not know whether DAT was initially inserted into the endosomes and then remained there or was inserted into the plasma membrane. Based on the results from previous investigations [43], we hypothesize that DAT was inserted into the plasma membrane in response to increased extracellular levels of DA in order to re-uptake the released neurotransmitter. In summary, our findings suggest that METH plays a role in trafficking of D2S and DAT in and out of DAergic terminals but affects the proteins differently; it triggers early decrease in D2S levels and late increase in DAT levels.

#### 2.3.3. Binge Methamphetamine Increases Immunoreactivity of D2S Receptor in Rat Striatal Synaptosomes Shortly after the Last Injection of the Drug

**Figure 5.**
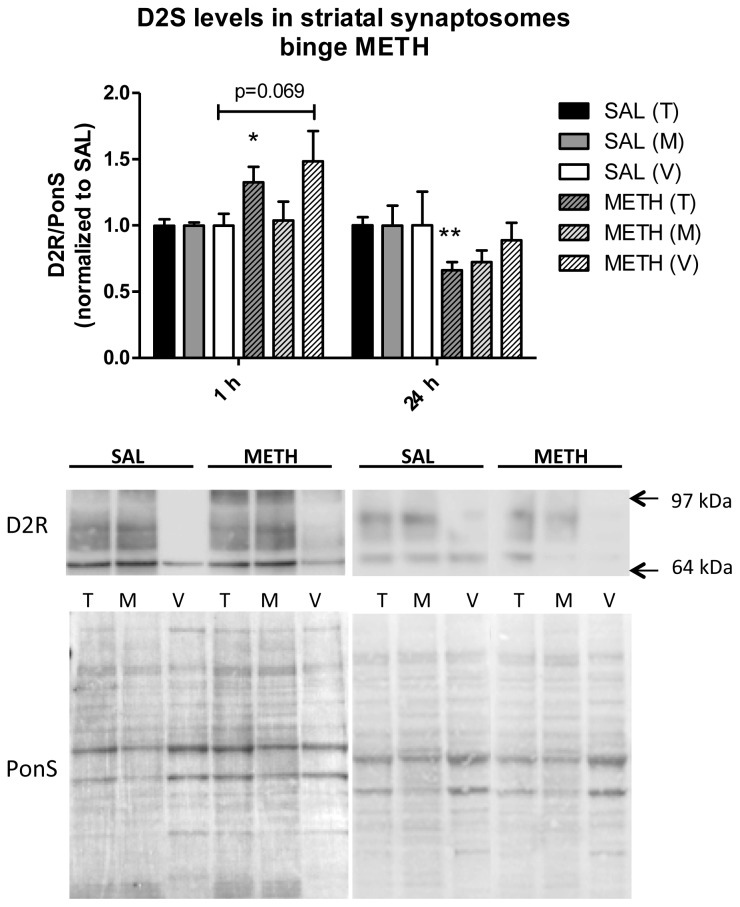
The effect of binge intraperitoneal (i.p.) METH on the immunoreactivity of pre-synaptic dopamine D2 autoreceptor (D2S) in striatal synaptosomal fractions. Binge METH (4 × 10 mg/kg, every 2 h, i.p.) increased D2 receptor immunoreactivity at 1 h in total synaptosomal fraction (+32%, * *p* < 0.05) and decreased D2 receptor immunoreactivity at 24 h in all three fractions, total, membrane-endosomal, and cytosol-vesicular fraction (−34% (** *p* < 0.01), −28%, and −11%, respectively). A trend for an increase in D2S immunoreactivity was observed in cytosol-vesicular fraction (+48%, *p* = 0.069). Abbreviations: M, membrane fraction; METH, methamphetamine; PonS, Ponceau S; SAL, saline; T, total fraction; V, vesicular fraction.

To study the effects of binge dose METH on the levels of D2S receptor in DAergic terminals, rats were administered binge METH (4 × 10 mg/kg, every 2 h, i.p.) or saline and sacrificed at 1 or 24 h after the last injection of the drug or saline. The striatal synaptosomal fractions (total, membrane-endosomal, and cytosol-vesicular) were examined for the levels of the receptor. As compared to saline controls, binge METH significantly increased immunoreactivity of D2S receptor in total synaptosomal fraction (+32%, * *p* < 0.05, Student’s *t*-test, *n* = 10 rats/group) at 1 h after the last injection of METH (Figure 5). There was also a trend for an increase in D2 receptor immunoreactivity in cytosolic-vesicular fraction (+48%, *p* = 0.069, Student’s *t*-test, *n* = 12–13 rats/group). At 24 h after binge METH, we found relatively small decreases in immunoreactivity of D2S in all synaptosomal fractions, *i.e.*, total, membrane-endosomal, and cytosol-vesicular fraction (−34%, −28%, and 11%, respectively) in METH-treated rats as compared to saline controls. The decrease in the total synaptosomal fraction was statistically significant (** *p* < 0.01, Student’s *t*-test, *n* = 9 rats/group). Cytosol-vesicular fractions from both saline- and METH-treated rats contained very low levels of D2 receptor, accounting for a rather high standard error of the mean. The antibody to the D2L receptor did not produce detectable immunoreactivity in any of the fractions at any time point (not shown).

Binge METH-induced increases in the immunoreactivity of D2S receptor in total synaptosomal and cytosol-vesicular fraction detected at 1 h after the last dose of the drug suggests decreased retrograde trafficking of the D2S receptor or its increased mobilization from a compartment outside the terminal (axon or cell body) to the cytosol accompanied by impaired targeting of the receptor to the plasma membrane. Alternatively, it suggests decreased degradation rate of the receptor and its accumulation in the cytoplasm. Oxidative stress can disrupt retrograde axonal transport of membrane proteins [25,59]. This finding together with the known ability of METH to cause oxidative stress [11] could account for our observations. An impaired targeting of the D2S to the plasma membrane can occur due to the lack of association with proper G proteins or lack of proper dimerization [35] as well as due to impaired interaction with other membrane proteins, such as adenosine A2 receptor or DAT. The adenosine A2A receptor resides on DAergic terminals in the striatum [53] and its binding to the D2S receptor can regulate its function, including regulation of D2S retention and mobilization to the plasma membrane [32,60]. The D2S receptor also forms a complex with DAT and regulates trafficking of the transporter [8,10]. Since DAT knock-out mice display lower levels and activity of the receptor [61] it is possible that DAT regulates D2S receptor trafficking as well.

METH or cytosolic DA might also regulate axonal transport of the D2S receptor. METH neurotoxicity is mediated by increases in both cytosolic and extracellular DA levels [62,63] and D2S receptor trafficking is regulated by extracellular DA. It can be hypothesized that D2S receptor trafficking might also be regulated by intracellular DA released by METH from the storage vesicles. Amphetamines have low affinity for the D2 receptor [64]. Consequently, METH binding to the D2S receptor might contribute to regulation of its trafficking. Binding of another psychostimulant, cocaine, to the D2 receptor affected insertion of the receptor into the plasma membrane [65].

The decreases in D2S immunoreactivity observed in all three fractions at 24 h after METH most likely represent a loss of DAergic terminals [11]. The decreases are smaller than those found at 5 d or 7 d after binge METH. METH-induced deficits in DAergic markers often do not reach the maximum at 24 h [37,38], which might have been the case in our study. The decreases might reflect, in part, a loss of D2S immunoreactivity due to increased degradation of the receptor, either as an adaptive response to DA release [50] or due to oxidative damage to the receptor [51].

#### 2.3.4. Binge Methamphetamine Has no Effect on the Immunoreactivity of DAT in Striatal Synaptosomes Shortly after the Last Injection of the Drug

**Figure 6.**
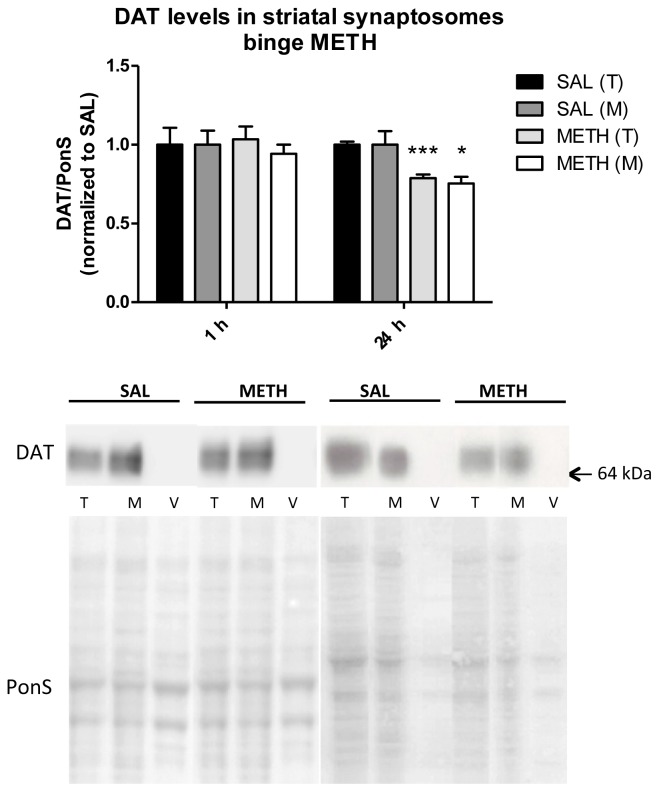
The effect of binge intraperitoneal (i.p.) METH on the immunoreactivity of dopamine transporter (DAT) in striatal synaptosomal fractions. Binge METH (4 × 10 mg/kg, every 2 h, i.p.) decreased DAT immunoreactivity at 24 h in total synaptosomal (−21%, *** *p* < 0.001) and membrane-endosomal (−25%, * *p* < 0.05) fraction. Abbreviations: M, membrane fraction; METH, methamphetamine; PonS, Ponceau S; SAL, saline; T, total fraction; V, vesicular fraction.

To study the effects of binge METH on the levels of DAT in DAergic terminals, rats were administered binge METH (4 × 10 mg/kg, every 2 h, i.p.) or saline and sacrificed at 1 or 24 h after the last injection of the drug or saline. The striatal synaptosomal fractions (total, membrane-endosomal, and cytosol-vesicular) were examined for the levels of the transporter. No signal for the DAT was detected in cytosol-vesicular fraction in saline- or METH-treated rats (Figure 6). As compared to saline controls, binge METH did not change immunoreactivity of DAT in total and membrane-endosomal fractions at 1 h (*p* > 0.05, Student’s *t*-test, *n* = 5–8 rats/group) but decreased DAT immunoreactivity in both fractions at 24 h (total: −21%, *** *p* < 0.001; membrane-endosomal: −25%, *p* < 0.05, Student’s *t*-test, *n* = 6 rats/group) (Figure 6).

Our finding of lack of change in DAT immunoreactivity in striatal synaptosomes at 1 h after METH agrees with an *in vivo* study that reported lack of change in DAT immunoreactivity in total synaptosomal fraction at 1 h after binge METH (4 × 7.5 mg/kg, s.c.) [23]. The data suggest that axonal transport of DAT is not increased or impaired by binge METH up to at least 1 h. The effects of binge METH on synaptosomal levels of DAT at time points between 1 and 5 h are currently investigated in our lab. At 24 h after binge METH, we found relatively small (~20%) but statistically significant decreases in immunoreactivity of DAT in both total and membrane-endosomal fraction, which most likely represent loss of DAergic terminals [11]. As discussed above, these decreases are smaller than those usually detected at 5 or 7 d after binge METH. The decreases in DAergic markers often do not reach the maximum at 24 h [37,38], which might have been the case in our study. The decreases might reflect, in part, a loss of DAT immunoreactivity due to oxidative damage [66,67] or changes in DAT synthesis and degradation rates after activation of D2 receptors by METH-released DA [68].

Longer exposure to METH (or DA) *in vitro* causes DAT internalization to intracellular compartments while shorter exposure does not. On the other hand, activation of D2 receptor mobilizes DAT to the plasma membrane as does direct D2S-DAT interaction [21]. Activation of D2 receptors by DA triggers their internalization [18]. We hypothesized that, in addition to affecting intracellular trafficking, METH also affects DAT and D2S axonal transport. So far, our data suggests that METH has different effects on DAT and D2S receptor axonal transport. Consequently, it is unlikely that the two proteins are transported together along the nigrostrial DA pathway and, therefore, it is unlikely that they interact in DAergic axons. They might interact in DAergic terminals.

### 2.4. Binge Methamphetamine Increases D2S Receptor-DAT Protein-Protein Interaction

**Figure 7.**
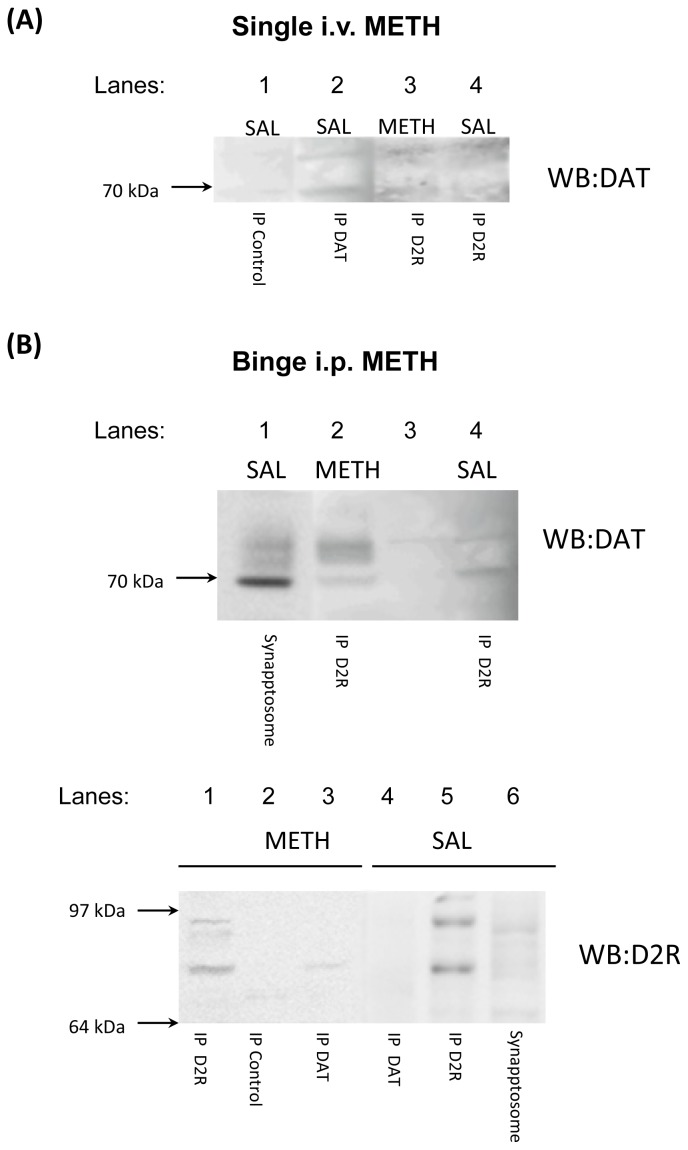
The assessment of dopamine pre-synaptic D2 autoreceptor-dopamine transporter (D2S-DAT) interactions after single and binge METH. Synaptosomes from saline- and METH-treated rats were subjected to co-immunoprecipitation using antibodies against DAT or D2 receptor (D2R). (**A**) Single METH (6 mg/kg, i.v.) did not cause any apparent changes to the levels of D2S-DAT complex (lane 3 *vs.* 4); (**B**) binge METH (4 × 10 mg/kg, every 2 h, i.p.) increased the levels of D2S-DAT complex (top: lane 2 *vs.* 4; bottom: lane 3 *vs.* 4). Controls: IP Control—negative control, Synaptosome—positive control. Abbreviations: IP, immunoprecipitation; i.p., intraperitoneal; i.v., intravenous; METH, methamphetamine; SAL, saline; WB, western blotting.

Dopamine D2S receptor and DAT are known to regulate each other’s function. The D2S receptor regulates DAT transporter activity via regulation of its trafficking [21]. Knock out of DAT lowers the levels and activity of D2S receptors relative to wild type controls [61]; therefore, DAT might regulate D2S receptor trafficking as well. To determine whether the D2 receptor forms a complex with the DAT *in vivo*, striatal synaptosomes from control and METH-treated rats (rats killed 30 min after single i.v. METH and rats killed 1 h after the last injection of binge i.p. METH) were subjected to co-immunoprecipitation with D2 receptor or DAT antibody followed by gel electrophoresis and western blotting with DAT or D2 receptor antibody, respectively. The 30 min end point instead of 1 h end point was chosen for single METH-treated rats because METH peaks in the brain ~30 min earlier after i.v. than i.p. administration [47]. The D2 receptor interacted with the DAT in saline animals (Figure 6A,B). As compared to respective saline controls, single dose METH did not change the levels of D2 receptor-DAT complex (Figure 7A) whereas binge METH increased the levels of D2 receptor-DAT complex in rat striatal synaptosomes (Figure 7B). The complex was formed between glycosylated species of the two proteins (>70 kDa).

Several studies have demonstrated functional interaction between D2S receptor and DAT, with D2S mediating increased expression of DAT at the plasma membrane via a phosphorylation pathway or physical interaction *in vitro* [10,69]. *In vivo*, physical interaction between D2S receptor and DAT was directly demonstrated in mouse striatum [27] and indirectly in rat striatum [28]. Our study directly demonstrated D2S-DAT interaction in rat striatum and showed that binge, but not single dose METH increased the levels of complex formed between the two proteins. *In vivo*, DAT was shown to form a complex with glycosylated D2 receptor from striatal membranes (>70 kDa) [70]. In agreement with the *in vivo* study, we observed the interaction between glycosylated species of the two proteins (70–90 kDa). Our data suggests the increased interaction between glycosylated D2S and DAT in DAergic terminals after binge METH. It can be hypothesized that the increase in D2S-DAT interactions occurred at the plasma membrane or in endosomes since no DAT was present in the cytosol.

*In vitro*, unglycosylated form of human D2S receptor was shown to form a complex with human DAT and mobilize DAT to the plasma membrane [10]. We did not observe increased levels of DAT in the membrane-endosomal fraction at 1 h after binge METH. The discrepancy might be due to different experimental conditions (*in vitro vs. in vivo* and no treatment *vs.* METH treatment), species differences (human *vs.* rat), intracellular compartment (cell body *vs.* terminal), and DAT glycosylation status (unglycosylated *vs.* glycosylated). Another study showed that amphetamine induces dimerization of the D2 receptor [28]. Similarly, binge METH induces oligomerization of DAT [71]. These data, together with the previous finding of decreased function of the DAT at 1 h after binge METH [72] and the current finding of increased levels of D2S-DAT complexes at the same time, suggest that binge METH leads to formation of D2S-DAT aggregates at the plasma membrane with consequent decrease in DAT function. The function of D2S is likely also decreased.

## 3. Experimental Section

### 3.1. Animals

Adult male Sprague-Dawley rats (Harlan, Indianapolis, IN, USA) (200–250 g) were pair-housed under a 12 h light/dark cycle in a temperature (20–22 °C) and humidity-controlled room. Food and water were available *ad libitum*. Temperature of the rats was measured via a rectal probe digital thermometer (Thermalert TH-8; Physitemp Instruments, Clifton, NJ, USA). All animal procedures were conducted in strict accordance with the National Institutes of Health (NIH) Guide for Care and Use of Laboratory Animals and approved by the Institutional Animal Care and Use Committee at Wayne State University (animal protocol #A 06-03-10, approved on 3 June 2010).

### 3.2. Administration of Methamphetamine

(+)-Methamphetamine hydrochloride (METH) (Sigma-Aldrich, St. Louis, MO, USA) or saline were administered to rats in two different regimens. In the binge regimen, METH (10 mg/kg, non-free base) or saline (1 mL/kg) was administered to adult male Sprague-Dawley rats every 2 h in four successive intraperitoneal (i.p.) injections. Rats were sacrificed by decapitation at 1 or 24 h after the last injection of METH or saline. This binge METH regimen is an established animal model of neurotoxicity to DAergic terminals [11]. Early neurotoxic events are most often monitored at 1 and 24 h after the last injection of METH. In order to generate data comparable to the results from previous studies, we chose the same time points. Hyperthermia is an important contributing factor in METH neurotoxicity; therefore, core body temperature was monitored throughout binge METH treatment, at 1 h after each injection. Core body temperature was also recorded after single METH injection, immediately before the sacrifice. Amphetamines and DA can affect trafficking of D2 receptor and DAT in bimodal manner, depending on the length of the exposure. To examine the effects of METH and METH-released DA over time, single high dose of METH (6 mg/kg, non free-base) or saline (1 mL/kg) was administered to rats intravenous (i.v.) via lateral tail vain. We chose i.v. and not i.p. route for administration of single dose of METH because the i.v. route allows very fast (2 min) delivery of METH to the brain thus allowing monitoring of rapid and transient trafficking of the membrane proteins [61]. We chose it also to decrease inter-individual variation, particularly at early time points; absorption from the peritoneal cavity may vary depending on the amount of food in the gut and needle placement. Bioavailability of METH after i.p. injection is ~60% of its availability after i.v. injection; therefore, we chose 6 mg/kg of METH for the i.v. route. The dose was well tolerated by the animals; no seizures were observed and the mortality rate was zero. The animals were euthanized via decapitation at 0 min after saline or 2 min, 10 min, 30 min, 1 h, 5 h, or 24 h after single METH. The dose of 6 mg/kg i.v. is a high METH dose; therefore, body core temperature was also monitored during its administration.

### 3.3. Tissue Content of Dopamine

Tissue content of DA in striata of rats sacrificed 7 d after METH was measured using a Shimadzu Prominence HPLC system with electrochemical detection (Shimadzu Scientific Instruments, Columbia, MD, USA). Frozen tissues were weighed and sonicated in 0.16 N perchloric acid. Precipitated proteins were removed by centrifugation at 14,000× *g* for 5 min at 4 °C. The resulting supernatants were collected and diluted 1:8 in 0.16 N perchloric acid. The mobile phase consisted of 50 mM sodium citrate, 50 mM sodium phosphate, 200 μM EDTA (Ethylenediaminetetraacetic acid), 1.5 mM heptanes sulphonic acid, and 14% methanol adjusted to pH 3.8. Concentration of DA was quantified by interpolating peak areas relative to those generated by a range of appropriate standards (Sigma Aldrich, St. Louis, MO, USA).

### 3.4. Preparation of Striatal Synaptosomal Fractions

Synaptosomal fractions (total, membrane-endosomal and cytosol-vesicular) were prepared as follows: harvested striata were manually homogenized in ice-cold 0.32 M sucrose solution using 2 mL dounce glass tissue grinder with Teflon pestle (Wheaton Industries, Inc., Millville, NJ, USA) and centrifuged (800× *g* for 24 min, 4 °C). Nuclei and other debris (P1) were discarded and the supernatant (S1) was again centrifuged (22,000× *g* for 17 min, 4 °C) to obtain total synaptosomal pellet (P2). The P2 was resuspended in distilled deionized water (ddH2O) and centrifuged (22,000× *g* for 17 min, 4 °C) to obtain cytosol-vesicular (S3) and membrane-endosomal (P3) fractions. Synaptosomal preparations were analyzed for protein concentration by BCA (bicinchoninic acid assay) protein assay using bovine serum albumin as the standard. All samples and resulting fractions were kept on ice at all times prior to analyses.

### 3.5. SDS-PAGE and Western Blotting

Synaptosomal fractions were subjected to sodium dodecyl sulfate-polyacrylamide gel electrophoresis (SDS-PAGE) under reducing condition (8% β-mercaptoethanol). 10–20 μg proteins were loaded per lane on 4%–12% Tris-Glycine gels. The amount of 20 μg protein/lane was loaded in most experiments. Sometimes, less protein was loaded per lane in cases where a sample or samples show low protein content and volume restriction (30 μL/well) did not allow 20 μg protein/lane. All proteins were transferred to PVDF (polyvinylidene difluoride) membranes and then blocked for 1 h at room temperature with 5% non-fat dried milk dissolved in TBST (Tris-buffered saline-Tween) (10 mM Tris, 150 mM NaCl, and 0.5% Tween-20). The membranes were then probed with either rabbit polyclonal D2 receptor primary antibody (1:1000, overnight at 4 °C) (AB5084P; EDM Millipore, Billerica, MA, USA), rabbit polyclonal primary D2L antibody (1:1000, overnight at 4 °C) (AB1792P, EDM Millipore) or goat polyclonal primary antibody DAT (1:1000; 1 h at room temperature) (SC1433; Santa Cruz Biotech, Santa Cruz, CA, USA). The incubations with primary antibodies were followed by washing and incubations with appropriate horseradish peroxidase (HRP)-conjugated secondary antibodies (1:10,000, 1 h at room temperature). Blots were developed using ECL detection and LAS4000 bioimager (GE Healthcare, Piscataway, NJ, USA). For standardization across blots, each blot contained all experimental groups. Ponceau S (P7170, Sigma-Aldrich) was used as a loading control in blots with synaptosomal fractions as binge METH causes partial redistribution of actin immunoreactivity from cytosol-vesicular to the membrane-endosomal fraction (not shown). Ponceau S and actin (1:1000; 1 h at room temperature) (AB1501R, EDM Millipore) were used as loading controls in blots with total synaptosomes and produced similar results. The western blot data was expressed as relative optical density units on each gel normalized to saline controls. This approach normalized differences in the development of the blot and across blots.

### 3.6. Co-Immunoprecipitation

Striatal synaptosomes were prepared from rats euthanized at 1 h after binge METH and from rats euthanized at 30 min after single dose METH or saline. The 30 min end point instead of 1 h end point was chosen for single dose METH-treated rats because METH peaks in the brain ~30 min earlier after i.v. METH than after i.p. METH [47]. Dynabeads (Life Technologies, Grand Island, NY, USA) were incubated in the presence of 2 μL of rabbit polyclonal D2 receptor primary antibody (AB5084P, 1:1000; EDM Millipore), polyclonal DAT primary antibody (SC1433; Santa Cruz Biotechnology Inc.), or radioimmunoprecipitation assay (RIPA) buffer (negative control) for 12 h at 4 °C, followed by the addition of synaptosomal fractions (200 μg of proteins in 100 μL of RIPA buffer) and second incubation (12 h at 4 °C). Following immunoprecipitation, DAT and D2 receptor were dissociated from the beads using SDS Tris-Glycine sample buffer (Bio-Rad, Hercules, CA, USA) and boiling (10 min at 100 °C), run on 4%–12% Bis-Tris gels under reducing conditions and subjected to western blot analysis using D2 receptor or DAT antibody and appropriate secondary antibodies, as described above.

### 3.7. Statistical Analysis

Data with two treatment groups were analyzed using Student’s *t*-test. Data from more than two treatment groups were analyzed using one-way ANOVA followed by Dunnett’s multiple comparisons *post hoc* test. Temperature data was analyzed using two-way ANOVA with repeated measures followed by Student-Neuman-Keuls *post hoc* test. All data are expressed as mean ± SEM. Statistical significance was set at *p* < 0.05.

## 4. Conclusions

Drug-exposed cultured DAergic cell lines or synaptosomes do not entirely mimic the intracellular or extracellular environment in the drug-exposed brain. In these studies, axonal transport, post-synaptic signaling of DA, and combined action of DA and METH are not factored in. Therefore, they are not ideal models to study axonal trafficking and interactions of D2S receptor and DAT, particularly in research on METH neurotoxicity, which affects terminals and not cell bodies in most species. In addition, studies in cell cultures do not take into account the fact that DAT traffics differently in different brain areas. Using an *in vivo* rat model, we have shown that METH has different effects on the levels of D2S receptor and DAT in striatal synaptosomes and that single dose METH affects D2S and DAT differently than binge METH. The data suggest that METH has different effects on axonal transport of the two proteins depending on the duration of the exposure with single dose METH (short exposure) decreasing D2S receptor and increasing DAT levels in DAergic terminals and binge METH (long exposure) reversing these effects. We have also shown that binge METH, neurotoxic to DAergic terminals, increases D2S-DAT interaction in DAergic terminals, most likely at the plasma membrane. As binge METH induces oxidative stress and oxidative damage to proteins, the most likely scenario involves oxidation-mediated impairment of D2S and DAT axonal transport, accompanied by accumulation of D2S in the cytosol and excessive formation of D2S-DAT complexes/aggregates at the plasma membrane. The results from our study provide potential additional mechanisms mediating METH neurotoxicity, namely impaired axonal transport and impaired D2S-DAT interactions in the nigrostriatal DA system.
